# Understanding Generalized Anxiety: Contributions from Phenomenology and Philosophy

**DOI:** 10.1192/j.eurpsy.2022.1727

**Published:** 2022-09-01

**Authors:** P.A. Gouveia

**Affiliations:** Local health Unit of Lower Alentejo, Psychiatry, LISBOA, Portugal

**Keywords:** phenomenology, generalized anxiety, philosophy, psychiatry

## Abstract

**Introduction:**

Anxiety is an ambiguous term, meaning an emotional state, a clinical symptom, a disorder, or a group of disorders. Anxiety is a normal feeling that arises when a person believes he is in danger from a threat or unidentified danger, ensuing with a state of alertness, arousal, and exploratory attention. Its distinction from neighbouring concepts, such as anguish, fear, worry, anxiety, panic, or uneasiness, is valuable but controversial.

**Objectives:**

Review and synthesize various contributions from phenomenology and philosophy to the understanding of what it is like to experience generalized anxiety.

**Methods:**

Selective review of the most prominent literature regarding anxiety psychopathology, namely that of Jaspers, Heidegger, López-Ibor, Sims, Berrios, Femi Oyebode, Pio Abreu, James Aho, Picazo Zappino and Gerrit Glas.

**Results:**

Jaspers described free-float anxiety as common and painful, floating and detached, as a feeling of misunderstood genesis, imposing despite the inapparent object, driving an inescapable need to provide some content to it, but also susceptible to insight by those who experience it. It can take a vitalized or primarily psychic form. Anxiety is closely related to the limits of the human being and to (hopelessness). For Heidegger, angst is the expression of authentic existence. López-Ibor considered anxiety and anguish nuances of the same experience, in both of which there is fear of the dissolution of the unity and continuity of the self (anguish). When what exists is not a fear, but only a threat, anxiety arises.

**Conclusions:**

Phenomenologically informed psychopathology is relevant for clinicians. Complementing neurosciences, each answers questions that the other cannot.

**Disclosure:**

No significant relationships.

